# Editorial: Luminescent Nanomaterials in Translational Medicine

**DOI:** 10.3389/fchem.2022.870300

**Published:** 2022-02-24

**Authors:** Benzhong Tang, Yan Yan, Lingming Zhang, Wenfu Zheng, Gang Chen, Ying Li

**Affiliations:** ^1^ Department of Chemistry, Institute for Advanced Study, The Hong Kong University of Science and Technology, Clear Water Bay, Kowloon, Hong Kong, China; ^2^ Centre for BioNano Interactions, School of Biomolecular and Biomedical Science, University College Dublin, Belfield, Ireland; ^3^ Key Laboratory of Molecular Target and Clinical Pharmacology, School of Pharmaceutical Sciences, Guangzhou Medical University, Guangzhou, China; ^4^ CAS Key Lab for Biological Effects of Nanomaterials and Nanosafety, National Center for NanoScience and Technology, Beijing, China; ^5^ Key Laboratory of Oral Biomedicine Ministry of Education, School and Hospital of Stomatology, Wuhan University, Wuhan, China; ^6^ Center for AIE Research, College of Materials Science and EngineeringShenzhen University, Shenzhen, China

**Keywords:** luminescent nanomaterials, theranostics (combined therapeutic and diagnostic technology), aggregation-induced emission luminogens, photodynamic therapy, photoacoustic imaging, chemical luminescence

Luminescent nanomaterials have attracted recent attention as imaging and therapeutic agents for biomedical science because they provide the advantages of non-invasion, high brightness, and easy biofunctionalization. This type of nanomaterial has become a powerful tool for visualizing tissues with cellular or sub-cellular resolution and mapping molecular events, and thus shows great potential in precision medicine that seeks to tailor specific therapies to individual patients. For application in precision medicine, the material interaction within living systems should also be considered and evaluated, which includes research on the material interface and photothermal effect in the physiological environment.

This Research Topic intends to present the cutting-edge of functionally developed luminescent nanomaterials with potential translational values, as well as the material interaction within living systems. The studies will contribute to the first-hand data in this field, providing a good reference for the clinical applications of the luminescent nanomaterials. This collection also encourages scientists to develop novel luminescent nanomaterials for the precise treatment of human diseases, and to explore the luminous mechanisms of these materials.

Aggregation-induced emission (AIE) luminogens have emerged as novel phototherapeutic agent with high photostability and capacity to generate reactive oxygen species. AIE photosensitizer emits intensely at high concentrations and overcome the aggregation-induced quenching effect of common fluorescence dyes. Yi et al. investigated the antibacterial effect of a AIE photosensitizer, TBP-1, against group B streptococcus (GBS) and the underlying mechanism. They found that TBP-1 killed GBS in either dark or light conditions. Interestingly, light exposure further enhanced the antibacterial effect of TBP-1 by inducing damage and morphological changes of membrane of GBS. He et al. summarized the recent advances on the molecular design of AIE photosensitizers and AIE dots-based probes including tetraphenylethylene and derivatives, triarylamine and derivatives and others. They also reviewed the reported applications of AIE photosensitizer for anticancer photodynamic therapy alone or in combination with chemotherapy, photothermal therapy and radiotherapy. The author discussed the challenges of this field including targeted delivery, treatment efficiency *in vivo*, development of novel AIE luminogens of photodynamic therapy and uncovering new mechanism of action of AIE photosensitizer.

Wu et al. utilized competitive host–guest interaction to detect cholesterol by chemical luminescence. This assay system consists of Fe_3_O_4_@SiO_2_ nanoparticles with the β-cyclodextrin (β-CD) decoration on their surface and ferrocene–Hemin complex that included through host–guest interaction with β-CD. Ferrocene-Hemin can be released upon the incubation with cholesterol through the competitive interaction between ferrocene–Hemin and cholesterol. Fe_3_O_4_@SiO_2_ nanoparticles containing cholesterol can be attracted to the bottom of assay tube by a magnet. The released ferrocene-Hemin thus catalyzes luminol/H_2_O_2_ reaction, the signal of which can be captured by a smartphone. This method has been successfully applied to quantify serum cholesterol after a simple liquid extraction process.

Feng et al. developed a strategy of antibacterial therapy that combines photodynamic therapy and sliver. They constructed a self-assembled nano-carrier consisting of photosensitizer (TCPP), anti-inflammatory agent (methotrexate), Poloxamer 407 and gallic acid. Silver element was further introduced through the reduction reaction of Ag^+^ that catalyzed by gallic acid. The synthesized nano-carrier showed antibacterial effect against *Escherichia coli* (*E. coli*) and *Staphylococcus aureus* (*S. aureus*) under the exposure of 620 nm laser.

Photoacoustic imaging (PAI) has emerged as a potential non-invasive diagnosis method in clinical applications. Huang et al. fabricated a cell membrane camouflaged nanoparticles to delivery DiR, a PAI contrast agent, for tumor imaging. DiR was encapsulated into poly (lactic-co-glycolic acid (PLGA) nanoparticles by emulsification and the synthesized nanoparticles were further coated with cancer cell membrane for homotypic targeting. This nanocarrier increased the circulation time of DiR in the blood and can be utilized for tumor imaging in mice.

In brief, this Research Topic presents some leading-edge researches on the evaluation of functionally developed luminescent nanomaterials, which shows great potential in the precise treatment of human diseases.

